# Clinical Feature, Therapy, Antimicrobial Resistance Gene Distribution, and Outcome of Nosocomial Meningitis Induced by Multidrug-Resistant Enterobacteriaceae—A Longitudinal Cohort Study From Two Neurosurgical Centers in Northern China

**DOI:** 10.3389/fcimb.2022.839257

**Published:** 2022-04-04

**Authors:** Guanghui Zheng, Yijun Shi, Yanfei Cao, Lingye Qian, Hong Lv, Lina Zhang, Guojun Zhang

**Affiliations:** ^1^ Department of Clinical Diagnosis, Laboratory of Beijing Tiantan Hospital and Capital Medical University, Beijing, China; ^2^ NMPA Key Laboratory for Quality Control of In Vitro Diagnostics, Beijing, China; ^3^ Beijing Engineering Research Center of Immunological Reagents Clinical Research, Beijing, China; ^4^ Department of Clinical Diagnosis Laboratory of Daqing Oilfield General Hospital Clinical Laboratory, Daqing, China

**Keywords:** MDRE, nosocomial meningitis, antimicrobial resistance genes, clinical feature, outcome

## Abstract

**Objectives:**

This is a comparative cohort study aiming to evaluate the mortality risk factors for patients with nosocomial meningitis (NM) induced by multidrug-resistant Enterobacteriaceae (MDRE) in China. The clinical features and therapies of patients and the resistance mechanisms of MDRE pathogens were also assessed.

**Methods:**

MDRE-NM patients from two neurosurgical centers in China from 2014 to 2019 were included in this study. Clinical features were extracted from the medical record databases of the two centers. The molecular mechanisms underlying the microbiological resistance mechanisms of each MDRE pathogen were determined, Kaplan–Meier survival analysis was conducted, and multivariable analyses were performed using a Cox proportional hazard model.

**Results:**

Ninety MDRE-NM patients were included in this study. *Klebsiella pneumoniae* accounted for the highest proportion of causative pathogens (46/90, 51.1%), and 40 causative pathogens (44.4%) were meropenem-resistant. blaKPC (27/40, 67.5%) was the predominant carbapenem resistance gene. Multivariate Cox analysis showed that external ventricular drainage (EVD) [hazard ratio (HR) = 2.524, 95% confidence interval (CI) = 1.101–5.787, p = 0.029] and a Glasgow Coma Scale (GCS) score ≤;8 (HR = 4.033, 95% CI = 1.526–10.645, p = 0.005) were mortality risk factors for patients with MDRE-NM. A total of 90.0%, 94.4%, and 97.8% of MDRE-NM patients received antibiotic prophylaxis (AP), antibiotic empirical therapy (AET), and antibiotic definitive therapy (ADT), respectively.

**Conclusions:**

NM caused by MDRE is an important sign of the failure of neurosurgery. MDRE possesses multiple drug resistance genotypes, and EVD and a GCS score ≤;8 are independent mortality risk factors for patients with MDRE-NM, which deserve the attention of microbiologists and neurosurgical clinicians.

## Introduction

Nosocomial meningitis (NM) is a serious neurosurgical complication that severely affects the survival of patients and the success rate of neurosurgery. According to literature reports, the mortality of NM patients is up to 15%–45% that of non-NM patients (Pintado et al., 2019).

Increasing antibacterial resistance in bacteria is a growing public health crisis that makes many medical care-associated infections difficult to treat with current antibiotics (Bielli et al., 2020; Liu et al., 2020). Multidrug-resistant Enterobacteriaceae (MDRE) is defined as Enterobacteriaceae resistant to at least three types of antibiotics (Teklu et al., 2019), including β-lactams, aminoglycosides, macrolides, and quinolones, among others. Because of their resistance to multiple drugs, clinicians are always limited in their choice of antibiotics. NM caused by MDRE usually leads to serious consequences for neurosurgical patients (Thatrimontrichai et al., 2021). Due to the complexity of neurosurgery and the existence of the blood–brain barrier, the mortality rate in patients with multidrug-resistant (MDR) bacterial meningitis is always higher than that in patients infected with wild-type strains (Liang et al., 2019).

This study focuses on the epidemiology and clinical features of the largest cohort of patients with MDRE-NM reported to date, with the aims of evaluating the clinical outcome of NM caused by MDRE and examining the risk factors for mortality in MDRE-NM patients. To the best of our knowledge, this is the first survival analysis on MDRE-NM patients conducted worldwide.

## Materials and Methods

### Study Settings and Ethics Statement

This retrospective study was approved by the ethical committees of Beijing Tiantan Hospital and Capital Medical University (KY-2021-079-02). All patients involved signed the informed consent form. This epidemiological and survival analysis study was conducted in two northern China neurosurgical hospitals, Beijing Tiantan Hospital and Capital Medical University and Daqing Oilfield General Hospital, from January 2014 to December 2019.

### Patient Inclusion and Clinical Data Collection

**Figure 1:**
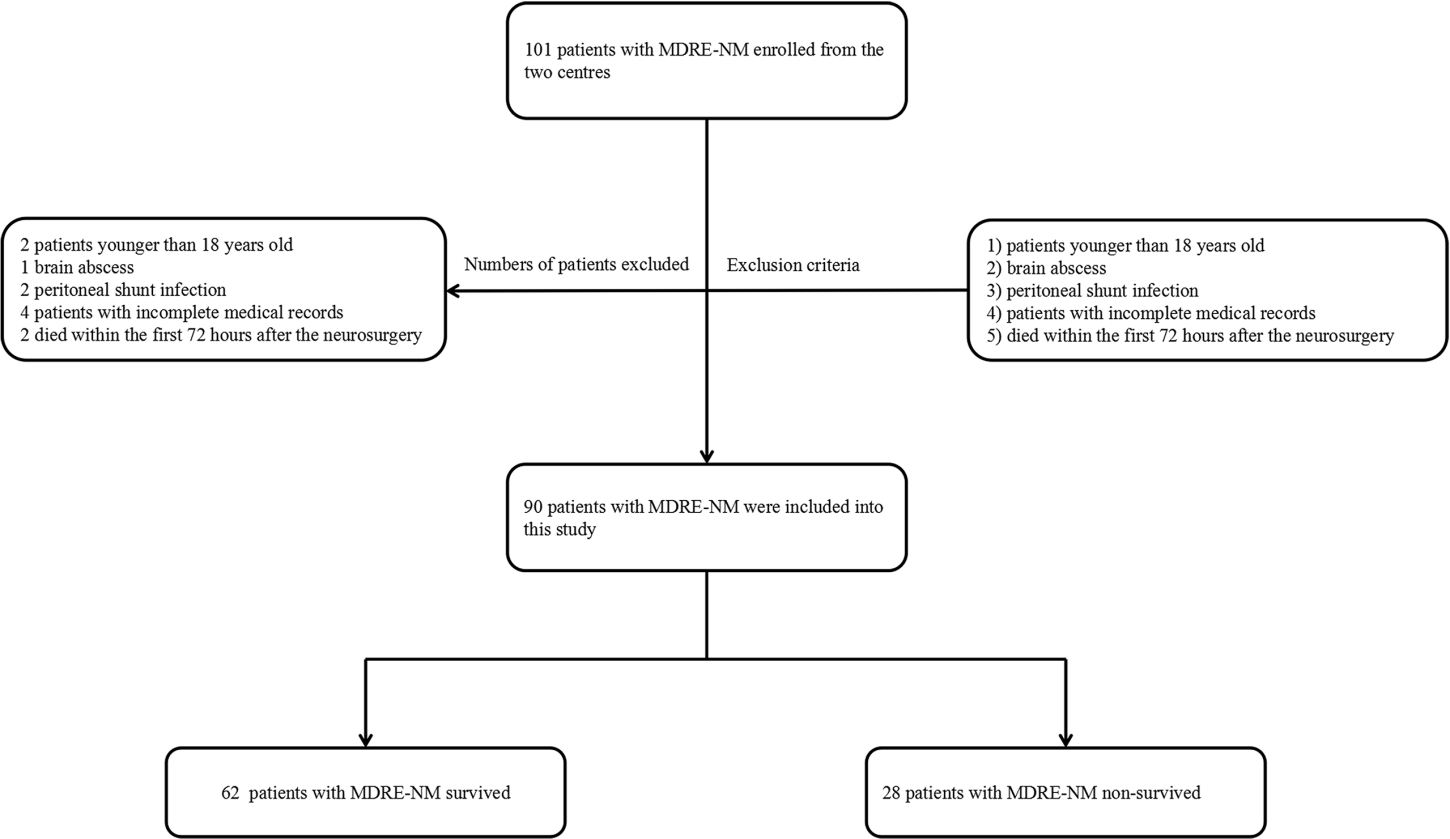
Flowchart of patient inclusion. *MDRE*, multidrug-resistant Enterobacteriaceae; *NM*, nosocomial meningitis.

MDRE is defined as Enterobacteriaceae resistant to three or more types of antibiotics, including β-lactams (meropenem and ceftriaxone), aminoglycosides (amikacin), quinolones (levofloxacin), tetracyclines, polymyxins B, sulfamethoxazole and trimethoprim (SMZ-TMP), and chloramphenicol (Magiorakos et al., 2012). The definition of NM provided by the U.S. Centers for Disease Control and Prevention (CDC) criteria was followed (Leverstein-Van Hall et al., 2010). Patients with a diagnosis of NM who were included in the study presented with bacterial proliferation in the cerebrospinal fluid (CSF) or at least one of the signs of meningeal irritation (headache, neck stiffness, or cranial nerve involvement for no other reason). These patients also displayed at least one of the following features: increased protein and/or decreased glucose level in the CSF, increased neutrophil count, positive CSF Gram stain, positive blood culture, positive antigen test in blood or CSF, or increased antibody titer against the pathogen. Neurosurgical patients with brain abscess, peritoneal shunt infection, incomplete demographic and clinical information and age younger than 18 years, and those who died within the first 72 h after the neurosurgery were excluded from the study. Patients were included if they were admitted into hospital after 48 h and underwent a neurosurgical procedure. When they identified with a CSF culture with MDRE in the study period, only the first isolate of MDRE in each patient was included. The flowchart and exclusion criteria are shown in **Figure 1**.

### Microbiology

MDRE pathogens isolated from the CSF underwent automated culture, bacterial identification, antimicrobial susceptibility tests (ASTs), and resistance gene screening. All bacterial cultures of the target patient CSF were carried out using a standard culture procedure, and 1–3 ml CSF was injected into aerobic automated culture bottles (bioMerieux, Marcy l’Etoile, France). Afterward, the bottles were transferred into BACT/ALERT^®^ 3D automated culture systems (bioMerieux, Marcy l’Etoile, France) until the bacteria grew to positivity. Then, positive cultures were subjected to standard microbial identification and AST procedures. The systems used for bacterial identification were VITEK MS (bioMerieux, Marcy l’Etoile, France; based on matrix-assisted laser desorption/ionization time-of-flight mass spectrometry) and VITEK-2 Compact (bioMerieux, Marcy l’Etoile, France; based on biochemical reaction). The identification procedures for VITEK-MS were as follows: a suspected Enterobacteriaceae colony was selected and smeared on the mass spectrometry identification plate, then 1 μl of the α-cyano-4-hydroxycinnamic acid (CHCA) matrix was add into the colony and allowed to stand at room temperature for 1 min. When crystals formed after volatilization of the matrix liquid, the identification plate was then placed in the VITEK-MS for detection. After 5 min, the identification results were obtained. The identification procedures for VITEK-2 Compact included selecting a suspected Enterobacteriaceae colony, employing 0.45% sodium chloride solution to prepare the bacterial suspension to 0.5 McFarland, and then inserting the GN microbial identification card (bioMerieux, Marcy l’Etoile, France) and transferring into the VITEK-2 Compact system for 24 h culture.

All Enterobacteriaceae identified by the methods described above were selected as the target bacteria. The broth microdilution method (MIC) and the Kirby–Bauer method were employed as the standard methods to perform the AST of Enterobacteriaceae. The breakpoints of each antibiotic followed the Clinical and Laboratory Standards Institute (CLSI) 2019 guidelines (CLSI, 2019). In MDRE pathogens, 21 resistance genes, including carbapenem (*bla_KPC_*, *bla_NDM-1_*, *bla_IMP_*, *bla_VIM_*, *bla_OXA_*_-23_, and *bla_OXA-66_*) extended-spectrum β-lactamases (ESBLs) (*bla_CTX_*_-_ *_M_* family, *bla_TEM_*, *bla_SHV_*, *bla_CYM-2_*, and *bla_DHA-1_*), aminoglycoside (*aadA1* and *aacC1*), tetracycline (*tetC*, *tetW*, and *tetQ*), quinolone (*qnrA* and *qnrS*), and polymyxin (*mcr-1*), were detected using the second-generation micro/nanofluidic chip platform-B (MNCP-II-B) based on the loop-mediated isothermal amplification method (LAMP) (Zheng et al., 2020). The extraction and test procedures were carried out according to the manufacturer’s instructions (Zhang et al., 2018).

### Patient Characteristics and Risk Factors

The daily medical records of the NM patients who qualified were extracted from the databases of the clinical infectious diseases, clinical neurosurgery, and clinical microbiology departments in these two centers. Of the medical record data, 21 characteristics were selected as the mortality risk factors for NM patients (**Table 1**). All of the patients were followed up to assess the development of NM in the first 30 postoperative days.

**Table 1:** Clinical characteristics and univariate analysis of survivors and non-survivors.

Characteristics	Total (*N* = 90)	Survivors (*n* = 62)	Non-survivors (*n* = 28)	*p*-value
Age (years)				0.877
Median	47	49	45	
IQR	31–60	31–59	34–60	
Male%	58	40	18	0.999
Hypertension	20	11	9	0.171
Diabetes mellitus	5	3	2	0.645
Fever (body temperature **>**38°C)	58	40	18	0.999
LD	37	23	14	0.259
EVD	46	28	20	0.024*
Long surgery duration (>180 min)	52	35	17	0.819
CSF leakage	19	12	7	0.583
Reoperation	25	16	9	0.614
AMV	38	16	22	<0.001*
LOS (days)				0.680
Median	27	25	30	
IQR	20–42	20–42	20–38	
Time of cure of infection				
Median	12	12	–	
IQR	7–18	7–18	–	
Surgical wound classification				0.999
Clean (I)	56	39	17	
Clean-contaminate (II)	34	23	11	
Craniotomy	62	41	21	0.467
GCS (≤8)	28	14	14	0.014*
Positive culture time				0.152
Median	7	7	11	
IQR	3.5–13	4–12	3–16	
ICU admission	43	21	22	<0.001*
Malignancy	52	30	21	0.499
Sepsis	39	20	19	0.003*
HAP	36	19	17	0.010*

IQR, interquartile range; LD, lumbar drainage; EVD, external ventricular drainage; LOS, length of hospital stay; AMV, assisted mechanical ventilation; GCS, Glasgow Coma Scale; ICU, Intensive care unit; HAP, hospital-acquired pneumonia.

*p < 0.05.

### Therapy

Antibiotics, which we used in the whole therapy procedure, were classified into three categories: antibiotic prophylaxis (AP), antibiotic empirical therapy (AET), and antibiotic definitive therapy (ADT). The three categories were defined as follows: patients who had AP received antibiotics 0.5 h before the neurosurgical operation; patients who had AET received antibiotics ahead of the AST result; and patients who had ADT received antibiotic therapy by AST guidance. In addition, the usage of high-grade antibiotics was evaluated.

### Statistical Analysis

To identify independent predictors for mortality, univariate analysis was employed to calculate the *p*-values for all variables, and Kaplan–Meier (K-M) survival analysis was performed for two groups for 30-day in-hospital survival. Any variables with *p* < 0.1 in the K-M survival analysis were included in the multivariate Cox proportional hazard model to analyze the independent MDRE-NM patient mortality risk factors. The results were expressed as the *p*-value, subdistribution hazard ratio (HR), and their 95% confidence intervals (CIs). Significance was defined as *p* < 0.05. WHONET 5.5 and SPSS (version 22; IBM, Armonk, NY, USA) were used for the statistical analysis.

## Results

**Figure 2:**
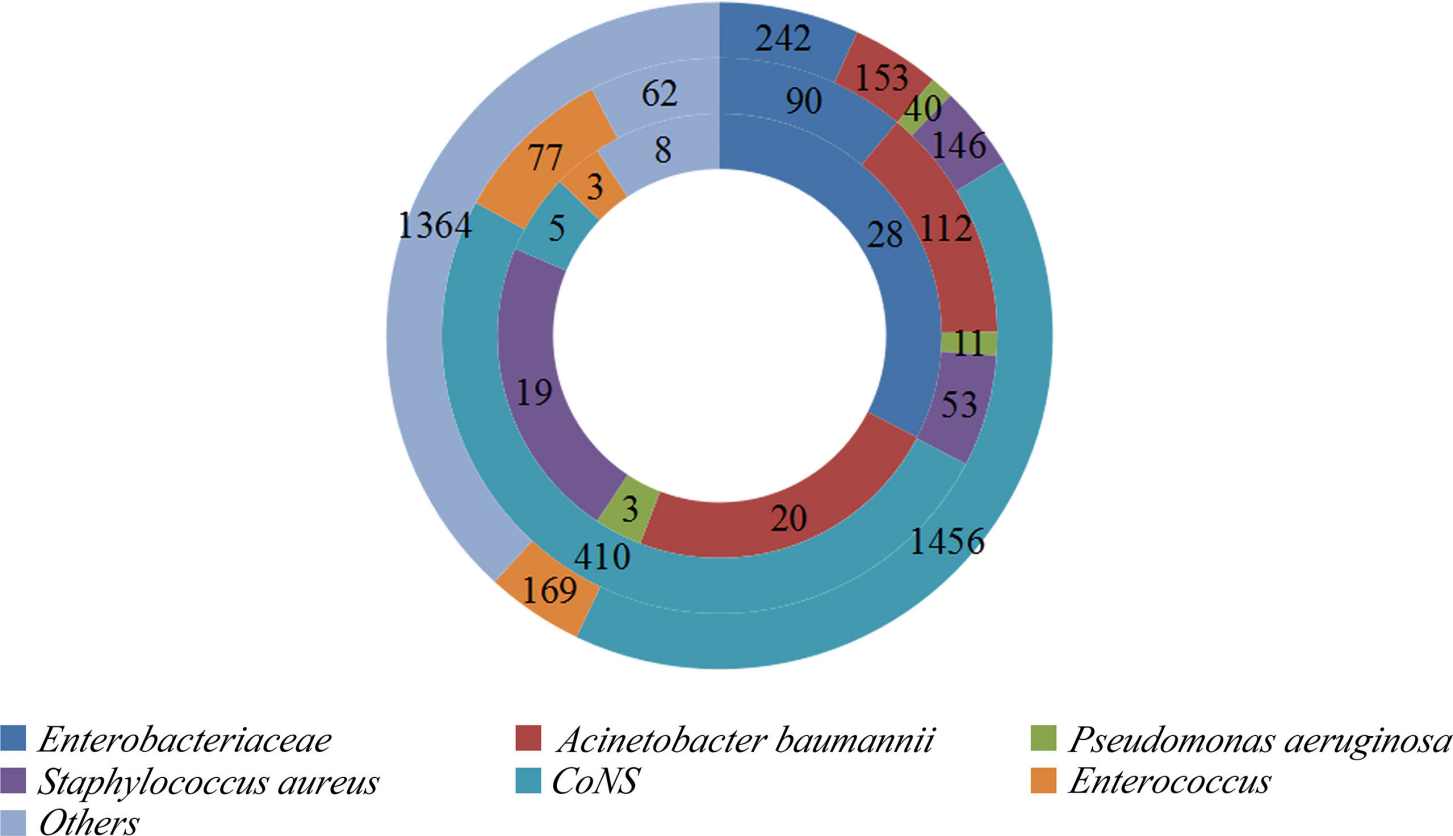
Distribution of bacteria isolated from the cerebrospinal fluid (CSF) of patients with nosocomial meningitis (NM).*Outer ring*: Distribution of bacteria. *Middle ring*: Distribution of multidrug-resistant (MDR) bacteria. *Inner ring*: Distribution of MDR bacteria causing NM mortality. *CoNS*, coagulase-negative staphylococci.

In total, 45,771 neurosurgery patients and 3,570 NM patients were observed in the two neurosurgical centers mentioned above. Among them, 22.8% (815/3570) of the cases were MDR patients; coagulase-negative staphylococci occupied the highest ratio. A total of 86 patients died of MDR NM, and besides MDRE, MDR *Acinetobacter baumannii* comprised the highest proportion. The distribution of these patients is shown in **Figure 2**. Of these patients, 242 cases of Enterobacteriaceae including 41.7% (101/242) of patients with MDRE-NM were recorded. Of them, two patients younger than 18 years, three patients hospitalized less than 7 days or without antimicrobial treatment, two patients who died within 7 days, and four patients with incomplete medical records (11 in total) were excluded from this study. Of the remaining 90 cases, 79 patients were from Beijing Tiantan Hospital and Capital Medical University (55 survival and 24 non-survival) and 11 were from Daqing Oilfield General Hospital (7 survival and 4 non-survival).

### Distribution, AST, and Genotyping of MDRE

**Figure 3:**
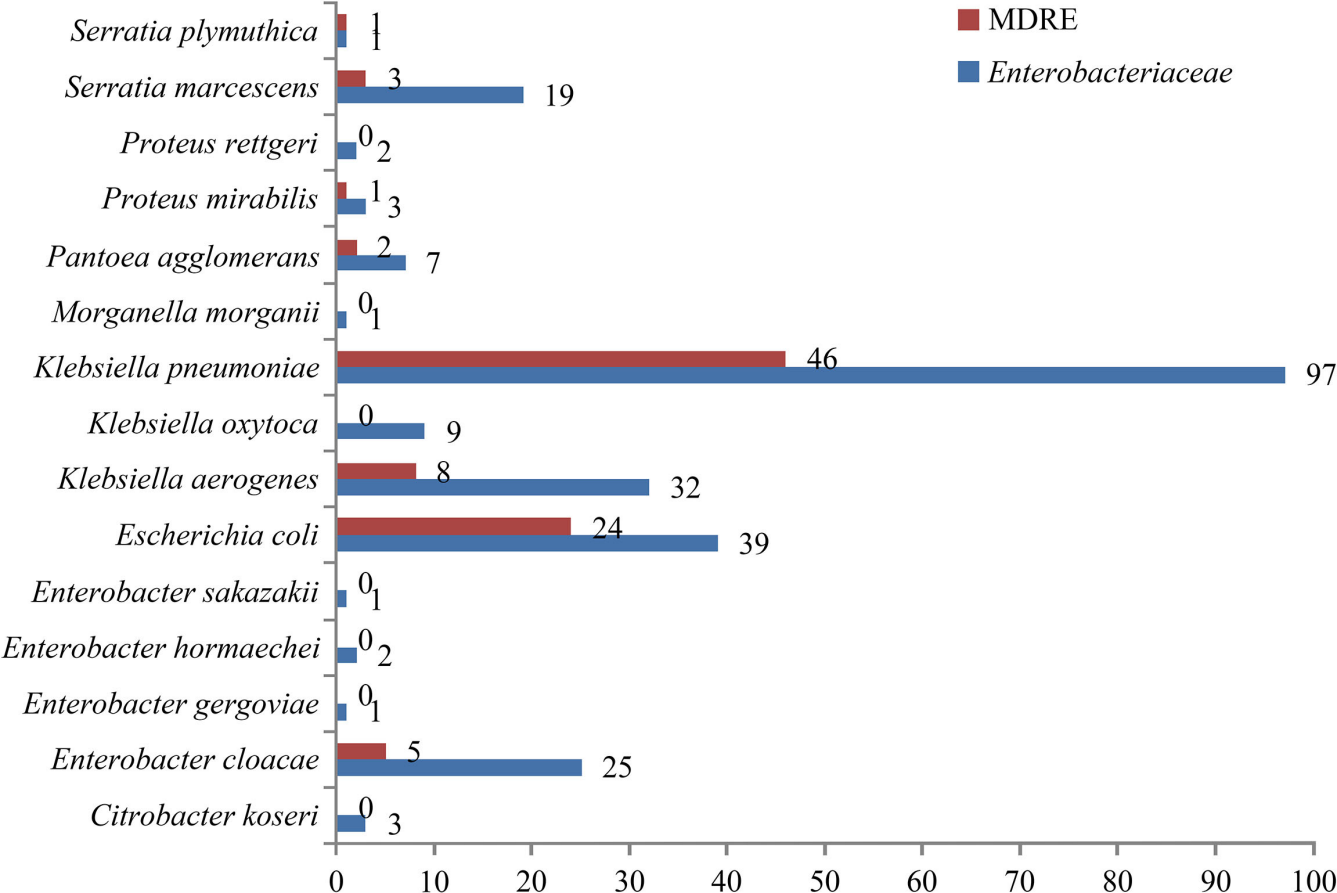
Number and distribution of NM caused by Enterobacteriaceae and MDRE. *MDRE*, multidrug-resistant Enterobacteriaceae; *NM*, nosocomial meningitis.

The isolated distributions of Enterobacteriaceae and MDRE are shown in **Figure 3**. *Klebsiella pneumoniae* was the MDRE with the highest incidence (46/90, 51.1%), followed by *Escherichia coli* (24/90, 26.7%), *Klebsiella aerogenes* (8/90, 8.9%), and *Enterobacter cloacae* (5/90, 5.6%).

**Figure 4:**
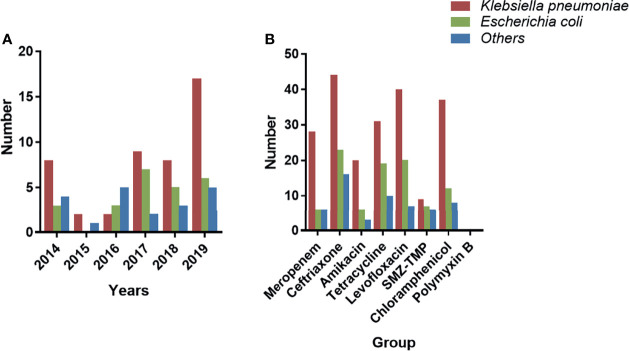
Distribution and AST of the MDRE group. **(A)** Year-by-year distribution of nosocomial meningitis (NM) caused by MDRE. **(B)** AST of MDRE. *AST*, antimicrobial resistance test; *MDRE*, multidrug-resistant Enterobacteriaceae.

**Table 2** shows the MDRE pathogen AST and genotyping results of the 90 NM patients. Among them, 40 (44.4%) were meropenem-resistant, and the prevalence of ceftriaxone resistance in the target MDRE was relatively high (83/90, 92.2%), whereas 32.2% (29/90) of the pathogens were found to be resistant to amikacin. No polymyxin B-resistant MDRE pathogens were found in this study, and the MDRE pathogens had a relatively lower frequency of resistance to SMZ-TMP (17/90, 18.9%). The resistance rates of MDRE to tetracycline, levofloxacin, and chloramphenicol were relatively similar, being 66.6% (60/90), 74.4% (67/90), and 63.3% (57/90), respectively. The year-by-year changes in the distribution of MDRE pathogens grouped by *K. pneumoniae* and *E. coli* are shown in **Figure 4A**; these MDRE pathogens accounted for the largest proportions in 2019. **Figure 4B** shows the AST results of the three types of MDRE pathogens, from which we found that *K. pneumoniae* dominated the majority.

**Table 2:** Antimicrobial susceptibility test (AST) and antimicrobial resistance genotyping of multidrug-resistant Enterobacteriaceae (MDRE) .

Antibiotic	Resistance	Genotype	*Klebsiella pneumoniae*	*Escherichia coli*	Others	Percentage (*n*)
Meropenem	44.4% (40/90)	*bla_KPC_*	23	3	1	67.5 (27/40)
		*bla_NDM-1_*	2	3	0	12.5 (5/40)
		*bla_IMP_*	2	1	0	7.5 (3/40)
		*bla_VIM_*	2	0	1	7.5 (3/40)
		*bla_OXA_*_-23_	2	3	1	15.0 (6/40)
		*bla_OXA-66_*	5	2	4	27.5 (11/40)
		ESBLs+*Ompk35*	21	3	2	65.0 (26/40)
Ceftriaxone	92.2% (83/90)	*bla_CTX_*_-_ *_M-1_*	3	5	2	12.5 (10/83)
		*bla_TEM_*	31	10	2	51.8 (43/83)
		*bla_CTM-M-9_*	17	18	6	49.4 (41/83)
		*bla_SHV_*	30	21	9	72.3 (60/83)
		*bla_CYM-2_*	3	0	0	3.6 (3/83)
		*bla_DHA-1_*	2	0	0	2.4 (2/83)
Amikacin	32.2% (29/90)	*aadA1*	18	2	0	69.0 (20/29)
		*aacC1*	1	0	1	6.9 (2/29)
Tetracycline	66.6% (60/90)	*tetC*	6	2	0	13.3 (*8/60*)
		*tetW*	2	2	1	8.3 (*5/60*)
		*tetQ*	0	0	0	0.0 (*0/60*)
Levofloxacin	74.4% (67/90)	*qnrA*	3	3	3	13.4 (9/67)
		*qnrS*	0	0	0	0.00 (0/67)
SMZ-TMP	18.9% (17/90)	–	0	0	0	–
Chloramphenicol	63.3% (57/90)	–	0	0	0	–
Polymyxin B	0.0% (0/90)	*mcr-1*	0	0	0	–

SMZ-TMP, sulfamethoxazole and trimethoprim. bla, β-Lactamase; KPC, Klebsiella pneumoniae carbapenemase; NDM, New Delhi metallo-β-lactamases; VIM, Verona integron-encoded metallo-β-lactamases; IMP, Imipenemase; OXA, Oxacillin-hydrolyzing carbapenemases; ESBLs, Extended spectrum-β-lactamases; OMPK, outer membrane protein K; TEM, Temoneira; CTM, cefotaxime; SHV, sulfhydryl reagent variable; CYM, cephamycins; DHA, Dhahran cephalosporinase; aad, aminoglycoside adenyltransferase; aac, aminoglycoside acetyltransferases; tet, tetracycline resistance gene; qnr, quinolone resistance gene; mcr, mobile colistin/polymyxin B resistance.

The meropenem-resistant MDRE pathogens had at least one carbapenem-related gene, and *bla_KPC_* (27/40, 67.5%) was the predominant gene. In addition, the ESBL-producing gene *Ompk35* (26/40, 65.0%) was also a more popular resistance gene, and *bla_OXA-66_* was expressed in 27.5% (11/40). In summary, *bla_SHV_*, *bla_TEM_*, and *bla_CTX-M-9_* were the three most popular ESBL-producing genes of MDRE. The whole genotype distributions are shown in **Table 2**.

### Outcome and Survival Analyses

**Figure 5:**
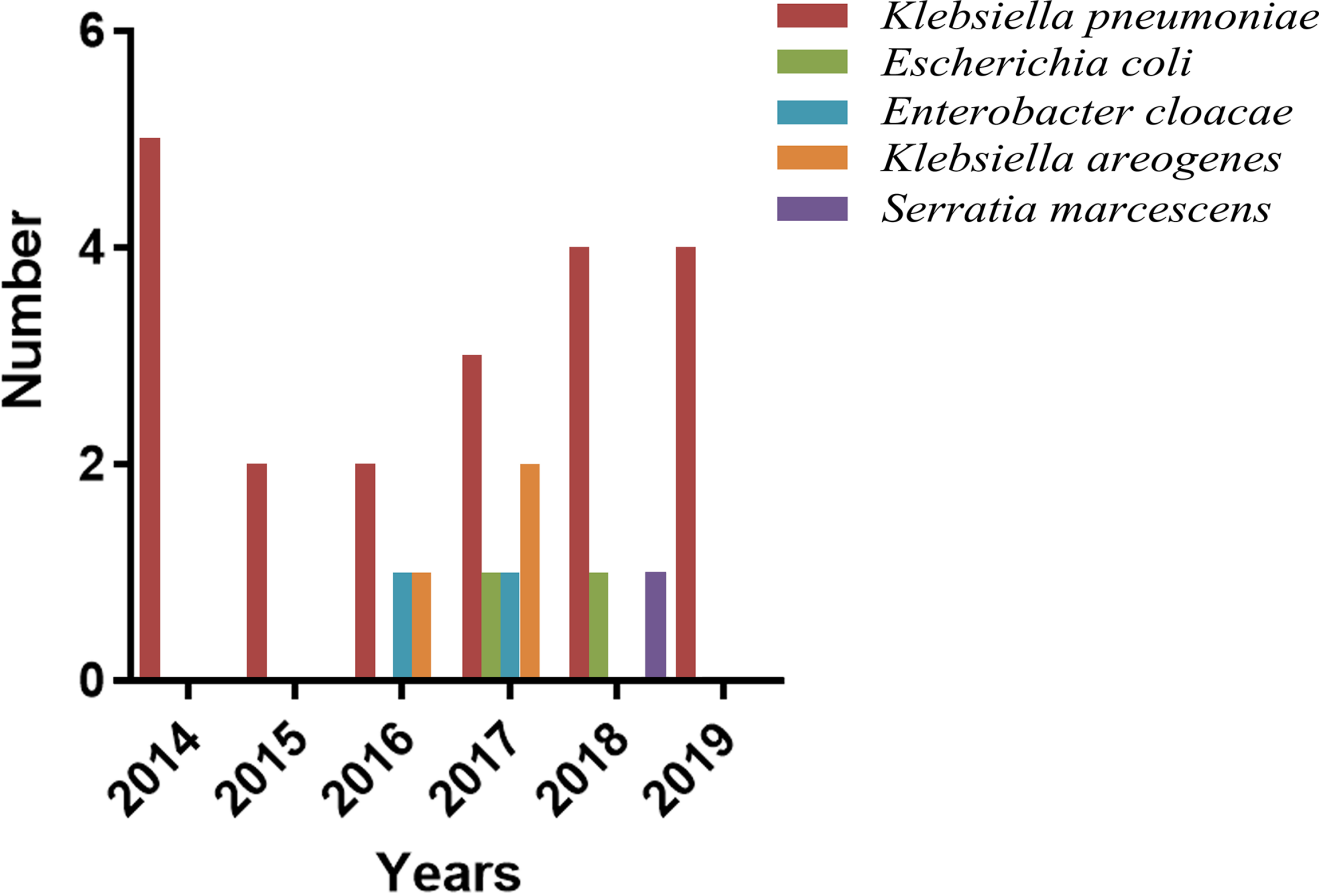
Year-by-year distribution of patients dying due to MDRE-NM. *MDRE*, multidrug-resistant Enterobacteriaceae; *NM*, nosocomial meningitis.

**Figure 6:**
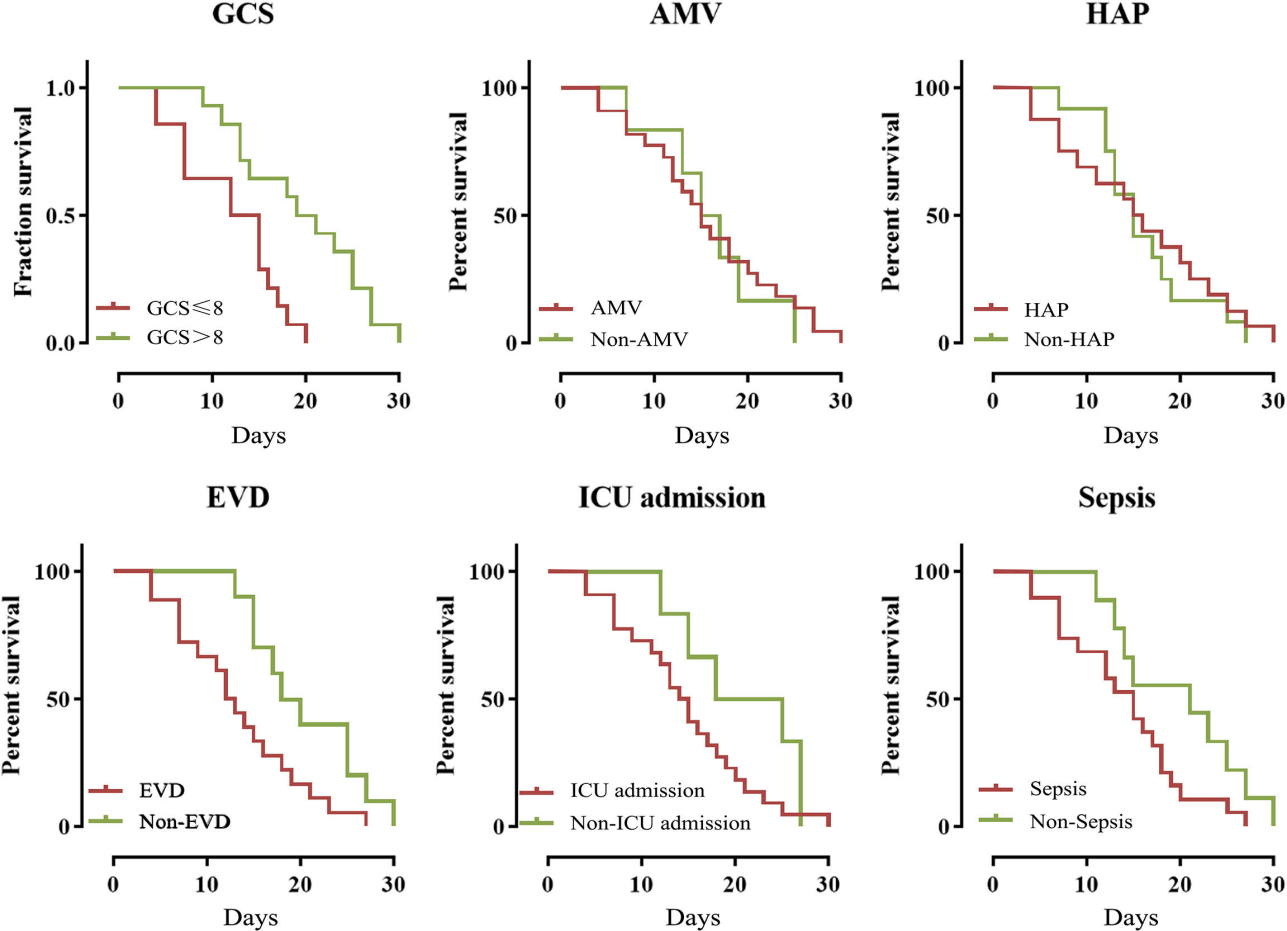
Kaplan–Meier analysis of the 30-day mortality of patients who experienced nosocomial meningitis caused by multidrug-resistant Enterobacteriaceae (MDRE-NM). *EVD*, external ventricular drainage; *GCS*, Glasgow Coma Scale; *ICU*, intensive care unit; *HAP*, hospital-acquired pneumonia; *AMV*: assisted mechanical ventilation.

The overall 30-day mortality was 31.1% (28/90). The year-by-year distribution of patients dying due to MDRE-NM is shown in **Figure 5**. From the figure, it can be seen that, in 2017, patients had the highest number of deaths due to MDRE-NM (7). Univariate analysis showed that the rates of EVD, assisted mechanical ventilator (AMV), GCS scores ≤;8, ICU admission, sepsis, and hospital-acquired pneumonia (HAP) were significantly different between patients in the survival and non-survival groups, and K-M survival analysis showed that EVD and the number of patients with a GCS score ≤;8 were significantly different between the two groups (*p* < 0.05). The results are shown in **Figure 6**. Multivariate Cox survival analysis showed that EVD (HR = 2.524, 95% CI = 1.101–5.787, *p* = 0.029) and a GCS score ≤8 (HR = 4.033, 95% CI = 1.526–10.645, *p* = 0.005) were independent mortality risk factors for patients with MDRE-NM (**Table 3**).

**Table 3:** Multivariate Cox analysis of the 30-day mortality for patients who experienced nosocomial meningitis caused by multidrug-resistant Enterobacteriaceae (MDRE-NM).

Characteristics	HR	95% CI	*p*-value
EVD	**2.524**	**1.101–5.787**	0.029*
AMV	0.461	0.118–1.803	0.266
GCS (≤;8)	**4.033**	**1.526–10.654**	0.005*
ICU admission	0.827	0.217–3.143	0.780
Sepsis	1.488	0.478–4.632	0.492
HAP	2.531	0.644–9.944	0.183

HR, hazard ratio; EVD, external ventricular drainage; AMV, assisted mechanical ventilation; GCS, Glasgow Coma Scale; ICU, intensive care unit; HAP, hospital-acquired pneumonia.

*p < 0.05. Bold values means that the two parameters are statistical difference by multivariate Cox analysis.

### Therapy

Of the 90 MDRE-NM patients included in this study, 90.0% (81/90), 94.4% (85/90), and 97.8% (88/90) received AP, AET, and ADT, respectively. Patients who received high-grade antibiotics accounted for the majority of those who had AET and ADT (95.3% *vs*. 96.6%). There was no significant difference in the prevalence of monotherapy, dual, and triple or more antibiotic therapy in patients of the ADT and AET groups (*p* = 0.274 and 0.089). The specific distributions of these antibiotic therapies are shown in **Table 4**.

**Table 4:** Comparison of antibiotic treatments for MDRE-NM patients.

Therapy	Total (*N* = 90)	Survivors (*n* = 62)	Non-survivors (*n* = 28)	*p*-value
Mono-antibiotic therapy (AET)	28 (32.9%)	20 (35.1%)	8 (28.6%)	0.247
Dual antibiotic therapy (AET)	44 (51.8%)	31 (54.4%)	13 (46.4%)	
Triple or more antibiotic therapy (AET)	13 (15.3)	6 (10.5%)	7 (25.0%)	
High-grade antibiotic therapy (AET)	81 (95.3%)	53 (93.0%)	28 (100.0%)	0.297
Mono-antibiotic therapy (ADT)	14 (15.9%)	13 (21.7%)	1 (3.6%)	0.089
Dual antibiotic therapy (ADT)	54 (61.4%)	35 (58.3%)	19 (67.9%)	
Triple or more antibiotic therapy (ADT)	20 (22.7%)	12 (20.0%)	8 (28.6%)	
High-grade antibiotic therapy (ADT)	85 (96.6%)	57 (95.0%)	28 (100.0%)	0.548
AP	81 (90.0%)	56 (90.3%)	25 (89.3%)	0.324

AP, antibiotic prophylaxis; AET, antibiotic empirical therapy; ADT, antibiotic definitive therapy.

*p < 0.05.

## Discussion

The emergence and spread of MDR pathogens, especially Enterobacteriaceae, is an important nosocomial health concern, making the improved molecular characterization and evaluation of the mortality risk factors for these strains vital. In this cohort study, the molecular characteristics and mortality risk factors for MDRE-NM were evaluated and compared with those of other types of MDR bacteria. The results revealed a high rate (101/242, 41.7%) of MDRE incidence among neurosurgical patients with NM, among which *K. pneumoniae* (46/90, 51.1%) was the most prevalent Enterobacteriaceae causing MDRE-NM. As evaluated by AST, third-generation cephalosporins (ceftriaxone) and carbapenems (meropenem) had high resistance rates of 92.2% and 44.4%, respectively. MDRE illustrated specific resistance genotypes for various antibiotics, among which *bla_KPC_* (27/40, 67.5%) and *bla_CTX-M-1_* (60/83, 72.3%) had the highest ratios. Multivariate Cox survival analysis showed that EVD and a GCS score ≤8 were independent mortality risk factors for patients with MDRE-NM.

Due to the limited choice of antibiotics, infections caused by MDRE can lead to more serious outcomes. In recent years, the increasing number of MDRE infections has brought great difficulties in clinical treatment. In our study, the proportion of *K. pneumoniae* was as high as 51.1%, which exceeded the proportion of Enterobacteriaceae reported in the literature (Balkhair et al., 2019), indicating that MDR *K. pneumoniae* caused more neurosurgical meningitis. *K. pneumoniae* possesses not only high drug resistance but also high virulence (Xu et al., 2019) and high invasiveness (Juan et al., 2019), and its infection mortality rate is also higher than those of other types of Enterobacteriaceae (Park et al., 2019). *E. coli* and *K. aerogenes* also accounted for certain proportions of the infections. These three types of Enterobacteriaceae account for 86.67% of all MDRE and are the most important pathogenic bacteria.

MDR is defined as bacterial resistance to at least three types of antibiotics. In this study, eight kinds of antibiotics, namely, meropenem, ceftriaxone, amikacin, levofloxacin, SMZ-TMP, tetracycline, chloramphenicol, and polymyxin B, were classified to screen MDRE pathogens. Target MDRE pathogens showed high resistance rates to meropenem (44.4%) and ceftriaxone (92.2%); moreover, resistance to tetracycline, levofloxacin, and chloramphenicol was relatively high. Nevertheless, the rates of resistance to amikacin, SMZ-TMP, and polymyxin B were low. All of the MDRE pathogens assessed were especially sensitive to polymyxin B, which is the last line of defense against MDR Gram-negative bacteria. A study reported that, for patients with severe multiresistant bacterial meningitis, polymyxin B can be injected intrathecally for emergency, which may be a better alternative for MDRE-NM treatment (Abad-Restrepo et al., 2018). However, treatment with polymyxin B causes several adverse reactions, such as nephrotoxicity (Zavascki and Nation, 2017), neuromuscular blockage (Myint et al., 2016), and respiratory depression (Hussein et al., 2010), so its application is limited to NM patients.

The presence of antibacterial resistance genes closely influences the choice of therapeutic drugs. For example, ceftazidime/avibactam has better activity against A- and D-type carbapenemase-producing bacteria, but is ineffective against the B type (such as *bla_VIM_* and *bla_IMP_*) (Zhanel et al., 2013). The distribution of carbapenemase-producing Enterobacteriaceae genes always varies; for example, the most prominent genotype in Europe is D-type *bla_OXA-48_* (Van Duin and Doi, 2017), the most prominent strain in India is *bla_NDM-1_* (Rahman et al., 2018), and the most prominent strain in China is *bla_KPC_* (Chi et al., 2019; Xu et al., 2019). In this study, the majority of the MDRE pathogens possessed more than one type of carbapenem resistance gene; nevertheless, *bla_KPC_* was the most frequent carbapenemase-producing mechanism. Previous studies have reported that undergoing loss of membrane porin protein and demonstrating modifications in permeability caused by efflux pump systems can significantly affect the resistance of bacteria to carbapenem (Petrosillo et al., 2013; Wassef et al., 2015). Our results confirmed that Enterobacteriaceae expressing the ESBL-producing gene+*Ompk35* comprised a large proportion; however, since the detection of *Ompk35* expression is not included in routine clinical laboratory test, ESBL + *Ompk35*, as a type of carbapenem resistance mechanism, also deserves the attention of neurosurgeons. Studies have confirmed that the predominant genotype of the MDRE varies in different regions and almost determines the phenotype of the MDRE. For example, an African study showed that *bla_SHV_* was the most frequent genotype, followed by *bla_TEM_*, *bla_CTX-M_*, and *bla_SHV_* (Nwafia et al., 2019). In a longitudinal analysis, *bla_CTX-M-15_* was the dominant ESBL-producing gene in all European countries, except Greece, where *bla_SHV_* was more common (Kazmierczak et al., 2020). Another study from the Netherlands, however, reported that *bla_CTX-M-1_* was the dominant gene, followed by *bla_CTX-M-15_* (Van Hoek et al., 2018). Screening for third-generation cephalosporin-related resistance genes in this study revealed that the three genotypes with the highest frequency of expression (*bla_TEM_*, *bla_CTX-M-9_*, and *bla_SHV_*) are all ESBL-producing resistance genes. Most likely because we investigated relatively few genes related to the production of the AmpC enzyme, fewer AmpC-related genes were detected. In fact, *K. pneumoniae* was the most common MDRE pathogen, and *bla_KPC_* was the predominant carbapenem resistance gene detected. In addition, univariate analysis showed that clinical factors such as AMV and ICU admission were significantly different in the two groups, which suggests that the prevalence of MDRE pathogens may be related to the clinical environment. Therefore, to evaluate the mechanism of resistance transformation, clinical molecular epidemiological approaches, such as multilocus sequence typing or pulsed-field gel electrophoresis, should be carried out (Xiong et al., 2021; Kim et al., 2022). For amikacin, tetracycline, and levofloxacin administration, we found that, although the MDRE pathogens showed resistance, we did not screen out the corresponding resistance genes. The reasons may be that our method for selecting resistance genes was insufficient and that testing for resistance genes was limited.

Survival analysis of MDRE-NM patients will help in their early treatment (Tewabe et al., 2018). Moreover, this analysis is a significant procedure to prevent the transfer of resistant bacteria among neurosurgical patients in the same ward. Control of the spread of resistant bacteria in the hospital will definitely help in maintaining patient health and safety. The prognosis of NM patients usually has poor outcomes, and prior studies reported that the mortality of NM patients with neurosurgery ranged between 20% and 78% (Karvouniaris et al., 2018). Several previous studies have identified mortality and infectious risk factors for meningitis, such as CSF leakage (Chen et al., 2020), coma (Olson et al., 2015), surgical intervention (Hanko et al., 2020), impaired consciousness (Mccormick et al., 2013), long surgery duration, AMV, delayed catheter removal even when clinically indicated, and non-sterile technique used during the surgery (O'Grady et al., 2011). In our study, the overall 30-day MDRE-NM mortality was 31.1%. EVD and a GCS score ≤;8 were associated with poor prognosis in patients with MDRE-NM. EVD is a common complication after brain surgery; nevertheless, the incidence of meningitis caused by EVD is high and increases the morbidity and mortality. Surveys such as those conducted by Lu et al. (2019) have shown that, in high-risk patients, the proportion of EVD-related infections may reach 22% (Arabi et al., 2005). EVD prolongs the length of hospital stay (LOS) of NM patients and increases their morbidity and mortality, increasing the hospitalization costs and even causing reoperations (Lyke et al., 2001; Eymann et al., 2008). The GCS provides an objective and reliable way of recording the conscious state of a patient. Generally, coma can be classified using GCS as severe, with GCS ≤;8; moderate, with GCS 9–12; and minor, with GCS >13 (Fu et al., 2020). Having a low GCS was a mortality risk factor and may have been predictive of the poor prognosis of MDRE-NM patients in our study. It is logical that low GCS scores indicate the severity of NM and coma, which easily leads to poor outcomes. Therefore, patients with EVD and with GCS ≤;8 require more extensive attention, such as the replacement of drainage catheters and the application of high-grade antibiotics, to obtain better treatment outcomes.

In the treatment of infectious disease, the application of combined antibiotics is of great significance; nevertheless, whether the combined application of antibiotics has a positive effect on the patient has not yet been determined. Tofas et al. reported that a combination of active drugs in carbapenemase-producing *K. pneumoniae* infection was associated with a lower mortality rate (Tofas et al., 2016). The results of this study showed that antibiotic combinations were more common in both AET and ADT. Regarding the outcomes of the patients, there was no significant difference in the effects of monotherapy and dual- or triple-combination drug therapy; this finding differs from those of previous reports. However, this result may be caused by the low number of included target patients. In addition, previous studies showed that the administration of polymyxin B is of great significance in the treatment of meningitis caused by MDR (Zhong et al., 2020), but in our hospital, it had fewer clinical applications.

There are several limitations to our study. Firstly, this was a retrospective study that evaluated patient mortality risk factors. This implies that the results of the study may not be representative of other studies. Secondly, the relatively small number of patients limited our epidemiological analysis. Thirdly, the number of drug resistance genes identified was relatively small, and many types of genes were not included. Further multicenter studies for mortality risk factor screening and more extensive evaluation of the resistance genes of the MDRE-NM causative pathogens may be required.

## Conclusion

In summary, to the best of our knowledge, this is the first report to conduct a two-center molecular and cohort study evaluating the mortality risk factors for MDRE-NM patients. Our study revealed that MDRE has multiple drug resistance genotypes, and EVD and a GCS score ≤;8 are independent mortality risk factors for patients with MDRE-NM, which deserve the attention of microbiologists and neurosurgical clinicians.

## Data Availability Statement

The original contributions presented in the study are included in the article/supplementary material. Further inquiries can be directed to the corresponding authors.

## Ethics Statement

This study was approved by the Ethical Committee of Beijing Tiantan Hospital and Capital Medical University (approval no. KY-2021-079-02).

## Author Contributions

GHZ, YS, YC: design and drafting of the article; data collection; and data analysis and interpretation. LQ: statistics. HL: MDR genotypes test. LZ and GJZ: conduct the whole study.

## Funding

This work was supported by the Beijing Hospital Authority Clinical Medicine Development of Special Funding (grant no. ZYLX202108) and Beijing Municipal Administration of Hospitals Incubating Program (grant no. PX2022021).

## Conflict of Interest

The authors declare that the research was conducted in the absence of any commercial or financial relationships that could be construed as a potential conflict of interest.

## Publisher’s Note

All claims expressed in this article are solely those of the authors and do not necessarily represent those of their affiliated organizations, or those of the publisher, the editors and the reviewers. Any product that may be evaluated in this article, or claim that may be made by its manufacturer, is not guaranteed or endorsed by the publisher.
